# Photoluminescence studies of a perceived white light emission from a monolithic InGaN/GaN quantum well structure

**DOI:** 10.1038/srep13739

**Published:** 2015-09-04

**Authors:** N. Ben Sedrine, T. C. Esteves, J. Rodrigues, L. Rino, M. R. Correia, M. C. Sequeira, A. J. Neves, E. Alves, M. Bockowski, P. R. Edwards, K. P. O’Donnell, K. Lorenz, T. Monteiro

**Affiliations:** 1 Departamento de Física e I3N, Universidade de Aveiro, Campus Universitário de Santiago,3810-193 Aveiro, Portugal; 2 IPFN, Instituto Superior Técnico, Campus Tecnológico e Nuclear, Estrada Nacional 10, P-2695-066 Bobadela LRS, Portugal; 3 Institute of High Pressure Physics, Polish Academy of Sciences, 01-142 Warsaw, Poland; 4 SUPA Department of Physics, University of Strathclyde, Glasgow, G4 0NG, Scotland, UK

## Abstract

In this work we demonstrate by photoluminescence studies white light emission from a monolithic InGaN/GaN single quantum well structure grown by metal organic chemical vapour deposition. As-grown and thermally annealed samples at high temperature (1000 °C, 1100 °C and 1200 °C) and high pressure (1.1 GPa) were analysed by spectroscopic techniques, and the annealing effect on the photoluminescence is deeply explored. Under laser excitation of 3.8 eV at room temperature, the as-grown structure exhibits two main emission bands: a yellow band peaked at 2.14 eV and a blue band peaked at 2.8 eV resulting in white light perception. Interestingly, the stability of the white light is preserved after annealing at the lowest temperature (1000 °C), but suppressed for higher temperatures due to a deterioration of the blue quantum well emission. Moreover, the control of the yellow/blue bands intensity ratio, responsible for the white colour coordinate temperatures, could be achieved after annealing at 1000 °C. The room temperature white emission is studied as a function of incident power density, and the correlated colour temperature values are found to be in the warm white range: 3260–4000 K.

GaN-based light-emitting diodes (LEDs) are attractive for many solid state lighting (SSL) applications. LEDs based on III-nitride low dimensional structures are capable of light emission from the ultraviolet to the red; however green and red emitting LEDs still suffer from low efficiencies^1^. LED-based light sources for general illumination have nowadays a tremendous impact in human life, residential and industrial sectors^2^. The global and societal technology development, including those related to energy saving, are promoting the SSL market towards a strong evolution in the next few years^2^.

Today, the best approaches for generating solid state white light are the use of colour mixing of a UV LED and a RGB phosphor^3^, RGB LEDs^4^, or a blue LED and a yellow phosphor^4^,^5^. For instance, in the last case, the so called pc-LEDs, white light is obtained using a two-step process: a commercial InGaN blue LED excites a Ce-doped YAG phosphor which emits yellow light (YL), then the mixture of blue light (BL) and YL is perceived as white. In addition to heating effects, phosphor-based white light emitters suffer from Stokes losses, non-radiative and optical losses, which present an important drawback of this technology^6^,^7^. For this reason, a lot of effort has been devoted to obtaining white emission from a monolithic system. To date, reports on achieving white emission using phosphor-free GaN-based systems have been based on: InGaN/GaN multiquantum well (MQW) structures with different In content and well thicknesses emitting in the primary colours (RGB)^8^,^9^,^10^, adding Si and Zn codopants to the InGaN/GaN structure^11^, In-rich InAlGaN/InGaN heterostructures^12^, InGaN/GaN MQWs grown on *c*-plane (0001) and on semipolar {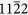} and {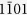} microfacets^13^,^14^, InGaN/GaN quantum dot^15^ and quantum well^16^,^17^ wavelength converter white LED heterostructures; and a defect-induced colour-tunable system based on blue InGaN/GaN MQW emission and broadband red emission in p-GaN^16^. Natural white (colour temperature ~6000 K) was demonstrated in thick InGaN nanodisks, grown on self-assembled GaN nanorod arrays, for which the numbers, positions, and thicknesses were tailored in order to get polychromatic nanodisk ensembles embedded vertically in the GaN nanorod p-n junction^18^. In the InGaN/GaN quantum dot wavelength converter white LED heterostructures grown by molecular beam epitaxy^15^, it was possible to achieve a 4420–6700 K correlated colour temperature (CCT) range, using an injection current density of 45 A.cm^−2^, by tuning the number of quantum dots, and the emission wavelength of the emission and converter dots. In this work, we demonstrate by photoluminescence analysis warm white light emission (3260–4000 K) from a monolithic InGaN/GaN single quantum well (QW)-based high quality structure, and show that the white emission persists even after annealing at 1000 °C and at high pressure, confirming the high stability of these structures after such post-growth conditions. Moreover, we show that the colour coordinate temperature can be controlled by the heat treatments.

## Experimental Details

The InGaN/GaN QW-based structure was grown by Metal Organic Chemical Vapour Deposition (MOCVD) on *c*-plane sapphire substrate. The structure is grown on a thick n-type GaN layer which is followed by the active region consisting of one 2.5 nm-thick InGaN QW with InN content of ~10%. An AlGaN electron blocking layer is used and the entire structure is capped by a 160 nm thick p-type InGaN layer with InN content of ~2%. Post-growth thermal annealing using high temperature and high pressure (HTHP) was performed at different temperatures: 1000 °C, 1100 °C and 1200 °C (denoted as HTHP-1000, HTHP-1100 and HTHP-1200, respectively), under a pressure of 1.1 GPa of N_2_ for 30 min. One as-grown sample was kept as a reference. X-ray diffraction rocking curve analysis of the 0004 and 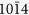 reflections showed values typical for state-of-the-art GaN samples with full widths at half maximum (FWHM) around 0.07°. No significant changes are observed after annealing revealing that structural properties, in particular the dislocation density, do not change significantly after annealing. Steady state photoluminescence (PL) spectroscopy was performed as a function of temperature (from 14 K to 300 K) using a cold finger He cryostat. The 325 nm line of a cw He-Cd laser (power density I_0 _< 0.6 W.cm^−2^) was used as excitation source, corresponding to an energy ~3.8 eV which is above the GaN and InGaN bandgaps. The sample luminescence was dispersed by a SPEX 1704 monochromator (1 m, 1200 gr.mm^−1^) and detected by a cooled Hamamatsu R928 photomultiplier. Photoluminescence excitation (PLE) and PL spectra were recorded at room temperature (RT) using a Fluorolog-3 Horiba Scientific modular apparatus with a double additive grating scanning monochromator (2 × 180 mm, 1200 gr.mm^−1^) in the excitation channel and a triple grating iHR550 spectrometer (550 mm, 1200 gr.mm^−1^) coupled to a R928 Hamamatsu photomultiplier for detection. A 450 W Xe lamp was used as excitation source. The measurements were carried out using a front face acquisition geometry, and the presented spectra were corrected for the spectral characteristics of the optical components and the Xe lamp.

## Results and Discussion

**Figure 1:**
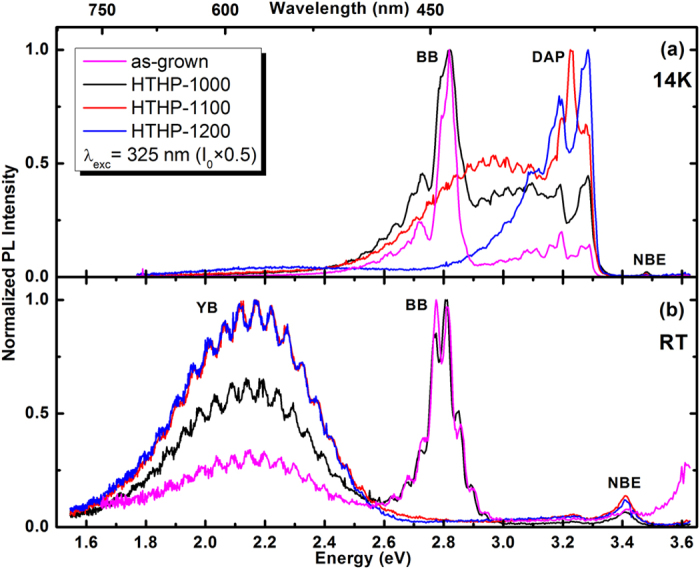
Normalized PL spectra of the as-grown, HTHP-1000, HTHP-1100 and HTHP-1200 samples at 14 K (**a**) and RT (**b**) obtained with 325 nm laser excitation.

Figure 1 shows normalized PL spectra of the as-grown, HTHP-1000, HTHP-1100 and HTHP-1200 samples at 14 K [Fig. 1 (a)] and RT [Fig. 1 (b)] obtained with 325 nm photon excitation and a power of I_0 _× 0.5. The oscillations in the PL spectra are related to Fabry-Perot optical interference within the structure. This Fabry-Perot effect results from the high refractive index contrasts especially between the layers and the substrate^19^ and modulates all the spectra. It is interesting to note that HTHP annealing at the different temperatures significantly changes the optical properties of the sample. At low temperature (14 K), the spectra of the as-grown and HTHP-1000 samples exhibit the QW blue band (BB) emission peaked at 2.8 eV (442 nm); its broadening (below 100 meV) is compatible with previously reported values^20^. For higher HTHP annealing temperatures (1100 and 1200 °C), the BB becomes undetectable indicating that such post-growth annealing temperatures might have affected the QW structure. On the high energy side, GaN near-band-edge (NBE) emission due to free and bound exciton transitions (at 3.48 eV), donor-acceptor pair (DAP) recombination and their phonon replicas^21^,^22^ are observed for all samples. Additionally, on the low energy side of the QW recombination, the broad yellow band (YB) is also identified with negligible intensity when compared with the BB intensity. It can be seen that the intensity ratio of the BB/DAP transitions is decreasing after HTHP annealing at 1000 °C. At RT, for the as-grown and HTHP-1000 samples, the BB persists at 2.8 eV, without any shift of the peak position compared to low temperature. In addition, for all samples, most of the DAP transitions thermally quench, and only the NBE (peaking at about 3.42 eV as expected from the shrinking of the GaN bandgap) and the broad YB centred at 2.14 eV (580 nm) are observed. Unstructured broad emission bands are well-known to occur in GaN samples due to the different nature of defects and typically involving deep defect levels^22^,^23^,^24^,^25^,^26^,^27^,^28^. In undoped and doped GaN layers, the most accepted models for the YB recombination involve a DAP or free-to-bound (e-A) transition related to the presence of the native defect V_Ga_ and its complexes (e.g. V_Ga_O_N_) in the nitride host^22^,^23^,^24^,^25^,^26^,^27^,^28^. It should be emphasized that, after HTHP annealing at 1000 °C, the intensity of the YB increases with respect to the as-grown sample, indicating that annealing in such conditions has promoted the contribution of the GaN deep defects to the emission. Since the samples annealed at 1100 °C and 1200 °C show only YB luminescence at RT, we will focus our analysis on the as-grown and HTHP-1000 samples.

**Figure 2:**
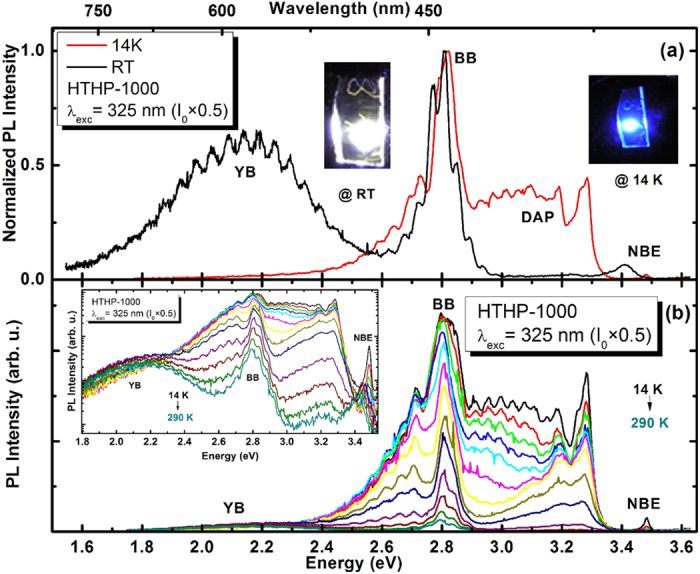
(a) 14 K and RT PL of the HTHP-1000 sample obtained with 325 nm laser excitation. Inset: photographs of the low and high temperature emissions (the bright circle in the center corresponds to the saturation of the camera’s detector due to the laser spot). (**b**) Temperature dependence PL spectra of the HTHP-1000 sample obtained with 325 nm laser excitation. Inset: PL temperature dependence in logarithmic scale for clarity.

In Fig. 2 (a), we present typical 14 K and RT normalized PL spectra of the HTHP-1000 sample obtained with 325 nm photon excitation and a power of I_0 _× 0.5. In the inset of Fig. 2 (a), we show photographs of the low and high temperature emissions. At 14 K, the sample emission is blue mainly due to the overlap of the BB and DAP transitions. However, interestingly at RT, white emission is clearly observed with the naked eye. Similar white emission is also observed under the same conditions for the as-grown sample (photograph not shown here, but will be discussed later). The detailed temperature dependent (14–300 K) PL spectra of the HTHP-1000 sample obtained with 325 nm laser excitation and a power of I_0 _× 0.5 are presented in Fig. 2 (b). The intensity of the PL spectra experiences a thermal quenching with increasing temperature between 14 K and RT (Fig. 2 (b) inset is a PL temperature dependence in logarithmic scale for clarity). At ~150 K, the intensity of the broad YB centered at 2.14 eV starts to influence the perceived colour, and persists up to RT to give a white emission when mixed with the BB. When increasing the temperature from 14 K to 300 K, the intensity of the blue band (peaked at 2.8 eV) decreases by a factor of twenty, but no shift of the peak position is observed. As the perceived white light results from an overlap of the two emitting centers, the absence of changes in the spectral shape and peak position are key properties to warrant the desired colour coordinates. In the UV range, most of the DAP transitions thermally quench, with only the NBE persisting at RT. This behaviour versus temperature is commonly observed in GaN-based structures.

**Figure 3:**
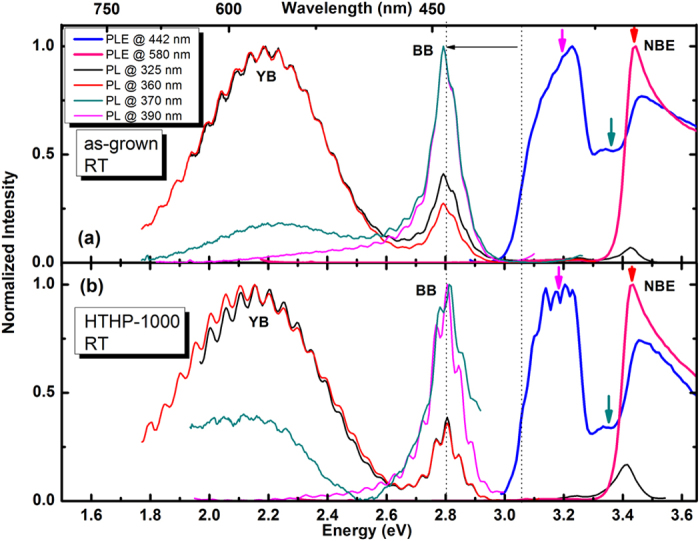
RT normalized PL (excited @ 325, 360, 370 and 390 nm) and PLE spectra (monitored @ 442 and @ 580 nm) of the as-grown (**a**) and HTHP-1000 (**b**) samples.

Figure 3 shows the RT PL spectra, measured under different photon energy excitations (325 nm (3.81 eV), 360 nm (3.44 eV), 370 nm (3.35 eV) and 390 nm (3.18 eV)), and PLE, monitored at the maxima of the YB (580 nm) and the BB (442 nm), of as-grown [Fig. 3 (a)] and HTHP-1000 [Fig. 3 (b)] InGaN/GaN QW structures. The different excitation energies used for the PL spectra are marked by arrows in the PLE spectra. It can be seen that both, as-grown and annealed samples present similar behaviour in PL and PLE. By pumping the samples above the GaN bandgap (325 nm) with the Xe lamp coupled with a monochromator, similarly to the results obtained using the He-Cd laser (Figs 1 and 2), the YB and BB emissions are observed, as well as the NBE. For PLE monitored at 442 nm (blue curve), the contributions from the InGaN QW and the GaN (barrier/template) are clearly distinguishable. For instance, by exciting at 360 nm (at the NBE of GaN), both BB and YB emissions can be recorded, with a maximum intensity obtained for the YB. However, the BB emission from the QW can be selectively excited using the 390 nm wavelength photons, as expected for a material with lower bandgap. An energy band-edge absorption of 3 eV could be obtained for the InGaN QW by assuming that the PLE and absorption spectra have the same shape^29^, and by fitting the absorption using the sigmoidal formula^30^. The high quality of the InGaN/GaN QW structure allows us to resolve, even at room temperature, the energy difference of ~200 meV (the so-called Stokes shift, denoted by a horizontal arrow in Fig. 3) between the band-edge absorption and the BB emission, which is often observed only at lower temperatures^31^,^32^.

**Figure 4:**
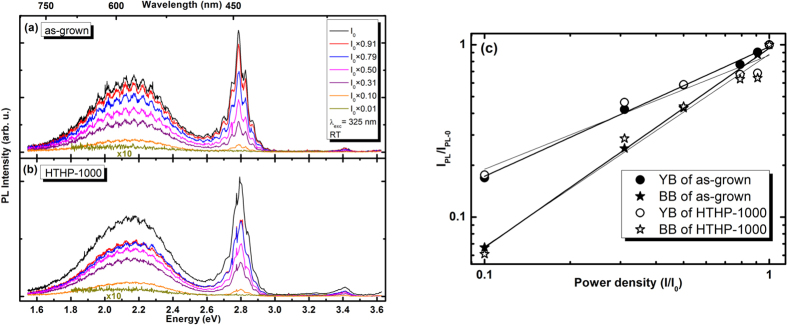
Laser power density dependence PL spectra at RT of the as-grown (**a**) and HTHP-1000 (**b**) samples obtained with 325 nm laser excitation. (**c**) Integrated PL intensity for YB and BB emissions (scatters) fitted to the power law 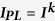 (lines).

In order to study in more detail the white emission observed in the as-grown and the HTHP-1000 samples, the RT PL response was measured as a function of the laser power density. The RT PL spectra are represented in Fig. 4 for the as-grown [Fig. 4 (a)] and the HTHP-1000 [Fig. 4 (b)] samples. It can be seen that no power-dependent shift in the BB and YB maxima is observed for either samples. The integrated PL intensity (*I*_PL_) for YB and BB emissions can be well fitted to a power law^33^: 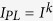, where *I* is the laser power density, and *k* the slope in a log-log plot [Fig. 4 (c)]. We have found a slope *k *≅ 1 for the BB emission of the as-grown and HTHP-1000 samples, consequently, the BB can be attributed to a free-exciton transition in the InGaN/GaN QW structure. For both samples, a slope of ~0.7 was determined for the YB emission, indicating free-to-bound or donor acceptor pair transitions involving a deep defect level in GaN accordingly with the aforementioned recombination model. Figure 4 (a,b) shows that increasing the power density over two decades results in a gradual increase of the BB PL intensity, while the increase of the power density up to *I*_*0*_ causes a decrease in the YB/BB intensity ratio from ~1.6 to ~0.6 for the as-grown sample, and from ~2.0 to ~0.7 for the HTHP-1000 sample. The distinct recombination models for the BB (excitonic in nature) and YB (e-A or DAP) PL bands are expected to respond differently to the excitation density. Particularly, for short lived excitonic well recombination such as green^34^,^35^ and blue^36^ emissions in similar structures, the PL intensity is expected to increase with excitation density almost linearly. However, for the long lived YB^28^, saturation effects are more pronounced even for low laser power densities. This explains the perceived change in the PL colour when observed with the naked eye, whereby the yellowish white emission turns to white when increasing the laser power density.

**Figure 5:**
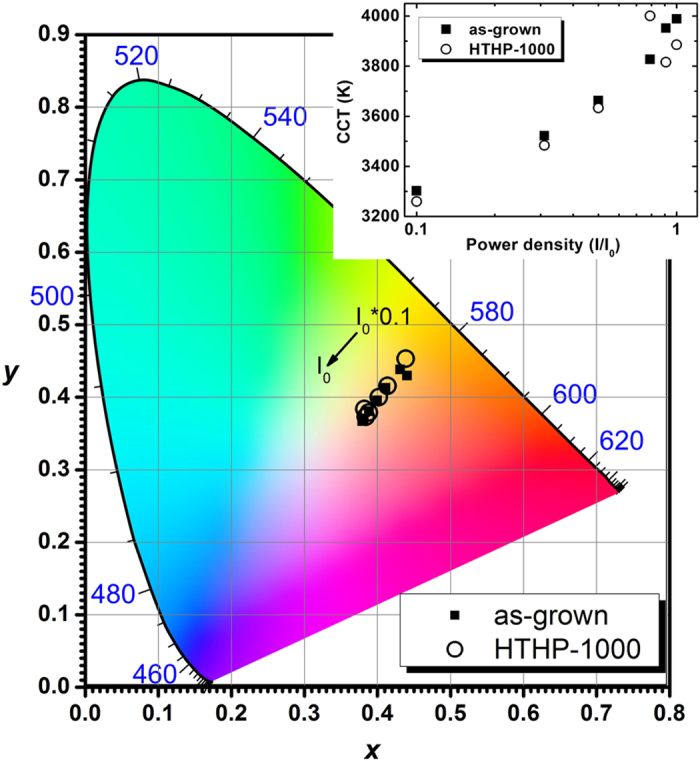
Chromaticity coordinates (CIE 1931) of the as-grown and HTHP-1000 samples for the different photon excitation power densities. Inset: correlated colour temperatures (CCT) of the perceived white photoluminescence, as function of power density.

Room temperature PL spectra versus laser power density for the as-grown and HTHP annealed at 1000 °C (HTHP-1000) QW structures were used to calculate the Commission Internationale de l’Eclairage (CIE 1931) chromaticity coordinates and the corresponding CCT (calculated after Ref. 37) of the perceived white light emission. The resulting chromaticity diagram is shown in Fig. 5, with the inset presenting the CCT as function of photon excitation power density. The CIE and CCT results are summarized in Table I. It can be seen from the chromaticity diagram, that when the laser power density increases, the CIE coordinates move from (0.37, 0.36) up to (0.43, 0.45), describing a white emission change from warm white to cooler white as expected due to the YB PL saturation. This can also be seen in the inset of Fig. 5 which shows an increase of CCT from 3260 up to 4000 K by increasing the laser power density. HTHP annealing studies on InGaN/GaN MQWs have already demonstrated that the structure and excitonic well recombination is stable after post-growth heat treatments. Particularly, in the case of the green QW emission, a high stability of the QW recombination was found after HTHP annealing at 1400 °C^35^. In the case of the investigated structures, the BB PL from the active layer still persists after a HTHP annealing up to 1000 °C. As such, the HTHP treatment constitutes an effective tool to tune the YB/BB PL intensity ratio in the QW structure.

## Conclusions

As-grown and HTHP annealed InGaN/GaN quantum well structures with active layer emitting in the blue region were analysed by optical techniques. Room temperature white light was perceived under 3.81 eV photon excitation, in the warm white range (3260–4000 K) for the monolithic InGaN/GaN structure due to the combination of the excitonic QW PL recombination and the yellow luminescence from GaN. Annealing the samples at 1000 °C and high pressure allows the control of the yellow/blue bands PL intensity ratio that is responsible for the white colour coordinate temperatures, which are dependent on the used photon excitation power density. Higher annealing temperatures completely quench the QW emission.

## Additional Information

**How to cite this article**: Ben Sedrine, N. *et al.* Photoluminescence studies of a perceived white light emission from a monolithic InGaN/GaN quantum well structure. *Sci. Rep.* **5**, 13739; doi: 10.1038/srep13739 (2015).

## Figures and Tables

**Table 1:** Chromaticity coordinates (CIE 1931) and correlated colour temperatures (CCT) of as-grown and HTHP-1000 structures under different He-Cd laser power densities.

	As-grown			HTHP-1000		
	Chromaticity coordinates			Chromaticity coordinates		
X	Y	CCT (K)	X	Y	CCT (K)	
1	0.37	0.36	3988	0.38	0.37	3885
0.91	0.38	0.37	3951	0.38	0.37	3816
0.79	0.38	0.38	3827	0.38	0.38	4000
0.50	0.39	0.39	3663	0.40	0.40	3633
0.31	0.41	0.41	3522	0.41	0.41	3483
0.10	0.43	0.43	3302	0.43	0.45	3259
